# Etifoxine improves sensorimotor deficits and reduces glial activation, neuronal degeneration, and neuroinflammation in a rat model of traumatic brain injury

**DOI:** 10.1186/s12974-016-0687-3

**Published:** 2016-08-26

**Authors:** Emmanuelle Simon-O’Brien, Delphine Gauthier, Véronique Riban, Marc Verleye

**Affiliations:** Pharmacology Department, Biocodex, Chemin d’Armancourt, 60200 Compiègne, France

**Keywords:** Traumatic brain injury, Etifoxine, Neuroinflammation, TSPO, Neurosteroids, Functional recovery, Cytokines, Astrogliosis, Neuronal degeneration, Microglia

## Abstract

**Background:**

Traumatic brain injury (TBI) results in important neurological impairments which occur through a cascade of deleterious physiological events over time. There are currently no effective treatments to prevent these consequences. TBI is followed not only by an inflammatory response but also by a profound reorganization of the GABAergic system and a dysregulation of translocator protein 18 kDa (TSPO). Etifoxine is an anxiolytic compound that belongs to the benzoxazine family. It potentiates GABAergic neurotransmission, either through a positive allosteric effect or indirectly, involving the activation of TSPO that leads to an increase in neurosteroids synthesis. In several models of peripheral nerve injury, etifoxine has been demonstrated to display potent regenerative and anti-inflammatory properties and to promote functional recovery. Prior study also showed etifoxine efficacy in reducing brain edema in rats. In light of these positive results, we used a rat model of TBI to explore etifoxine treatment effects in a central nervous system injury, from functional outcomes to the underlying mechanisms.

**Methods:**

Male Sprague-Dawley rats received contusion (*n* = 18) or sham (*n* = 19) injuries centered laterally to bregma over the left sensorimotor cortex. They were treated with etifoxine (50 mg/kg, i.p.) or its vehicle 30 min following injury and every day during 7 days. Rats underwent behavioral testing to assess sensorimotor function. In another experiment, injured rats (*n* = 10) or sham rats (*n* = 10) received etifoxine (EFX) (50 mg/kg, i.p.) or its vehicle 30 min post-surgery. Brains were then dissected for analysis of neuroinflammation markers, glial activation, and neuronal degeneration.

**Results:**

Brain-injured rats exhibited significant sensorimotor function deficits compared to sham-injured rats in the bilateral tactile adhesive removal test, the beam walking test, and the limb-use asymmetry test. After 2 days of etifoxine treatment, behavioral impairments were significantly reduced. Etifoxine treatment reduced pro-inflammatory cytokines levels without affecting anti-inflammatory cytokines levels in injured rats, reduced macrophages and glial activation, and reduced neuronal degeneration.

**Conclusions:**

Our results showed that post-injury treatment with etifoxine improved functional recovery and reduced neuroinflammation in a rat model of TBI. These findings suggest that etifoxine may have a therapeutic potential in the treatment of TBI.

## Background

Traumatic brain injury (TBI) is a leading cause of mortality and morbidity and can result in long-term physical, behavioral, and cognitive deficits. These deficits are due to both primary and secondary injuries. The primary injury occurs at the moment of TBI impact, with disruption of blood brain barrier and blood vessels that contribute to edema formation [1]. This primary injury precedes several downstream events contributing to secondary injury cascade of cellular, molecular, and metabolic pathological events, such as activation of brain-resident microglia and astrocytes, production of cytokines and chemokines, or recruitment of peripheral immune cells into the brain [2–4]. TBI is also followed by a profound reorganization of the GABAergic system and a dysregulation of the mitochondrial 18kDa translocator protein (TSPO) levels [5]. These events may lead to acute and chronic cell death and contribute to functional impairments [6, 7]. The onset of secondary injuries can be delayed for minutes to hours and persist for weeks to months [8, 9].

Etifoxine (EFX) ((2-ethylamino-6-chloro-4-methyl-4-phenyl-4H-3,1-benzoxazine hydrochloride) is an anxiolytic compound that belongs to the benzoxazine family. Its anxiolytic and anticonvulsant properties have been shown in rodents [10, 11], and it is effective in the treatment of adjustment disorders with anxiety in humans [12–14]. Etifoxine binds directly to the GABA_A_ receptor, acting as a positive allosteric modulator, thus potentiating GABAergic synaptic transmission [10, 15–17]. EFX also activates TSPO [18] which plays an important role for the synthesis of neurosteroids [19]. Etifoxine was indeed shown to inhibit the binding of the selective TSPO ligand [3H]PK11195. In addition, i.p. administration of EFX increased plasma and brain concentrations of progesterone, pregnenolone, 5α-dihydroprogesterone, and allopregnanolone [18, 20]. The formation of these neurosteroids might result in a brain region-specific enhancement of GABAergic neuronal inhibition [21]. In the central nervous system (CNS), TSPO is generally expressed in both microglia and in reactive astrocytes [22]. Although TSPO level in the brain is low, it increases after brain injury [5, 22] and is thus a marker of brain injury and repair [5, 23–25]. Emerging evidence suggests that administration of TSPO selective ligands may be a therapeutic tool in the treatment of inflammatory conditions [26] and might be neuroprotective [27–29]. Some studies showed that EFX displays potent regenerative and anti-inflammatory properties, promotes functional recovery in experimental models of traumatic peripheral nerve injury, and reduces brain edema in rats [30–32]. To investigate further the potential neuroprotective and anti-inflammatory effect of EFX, we tested this compound in a model of TBI induced by controlled cortical impact (CCI) [33, 34] in male adult rats. This model has numerous advantages such as the possibility to control deformation parameters, including duration, depth, and velocity of impact.

## Methods

### Animal subjects

All experiments were performed on male Sprague-Dawley rats weighing 250–300 g at the time of injury (Janvier laboratories, Le Genest Saint Isle, France). Rats were group-housed with *ad libitum* access to food and water in a temperature-controlled (22 ± 2 °C) and humidity-controlled (55 ± 10 %) environment. Lights were on a 12-h light/dark cycle (lights on at 7:00 a.m). Experiments were carried out in strict accordance with the European Community regulations for animal use in research (2010/63/EU directive), and all protocols were approved by the Local Ethical Committee (C2EAn°72).

### Drugs

Etifoxine (batches 562 and 653, Biocodex, Gentilly, France) was suspended in 0.9 % saline containing 1 % Tween 80 (vol/vol). This compound and its vehicle were administered intraperitoneally (i.p.) in a volume of 5 mL/kg of bodyweight.

### Traumatic brain injury procedure

Rats were subjected to TBI using a controlled cortical impactor (Impact One™, Leica Microsystem, Illinois, USA). Following deep anesthesia using isoflurane (induction 4 %, maintenance 2 %), rats were stabilized in a stereotaxic frame (David Kopf Instruments, California, USA) and placed on a heated pad. After exposing the skull, a 4-mm diameter circular craniotomy was performed using an electronic hand drill. Rats received contusion injury centered 2 mm lateral to the bregma over the left sensorimotor cortex with a velocity of 3.0 m/s, a depth of 1.5 mm, and a duration of 0.5 s [34–38]. The impactor tip was angled 8° vertically to maintain a perpendicular position in reference to the tangential plane of the brain convexity at the impact surface. A small quantity of saline solution at room temperature was directed at the site of drilling to prevent thermal injury [39]. After the impact, the scalp was sutured closed. Sham animals received craniotomy but no impact from the CCI device. All animals were closely monitored post-operatively with weight and health surveillance.

### Experimental design

This study was divided in two parts. In the first part, we wanted to assess the effects of EFX treatment on sensorimotor deficits induced by CCI in rats. To this aim, we administered EFX at the anxiolytic-like effective dose of 50 mg/kg, i.p. [10], or its vehicle, 30 min after TBI (*n* = 10 for TBI-vehicle group, *n* = 8 for TBI-EFX group) or sham surgery (*n* = 9 for sham-vehicle group, *n* = 10 for sham-EFX group), then daily after behavioral testing during 1 week. The second part consisted in studying the effects of EFX treatment on neuroinflammation markers induced by TBI in our rat model (*n* = 5/group). The critical time-points to study were chosen based on the behavioral results.

#### Effects of EFX treatment on sensorimotor performances

##### Bilateral tactile adhesive removal test

This test was performed on days 2, 5, and 7 post-CCI in order to test the forepaw sensitivity as well as motor impairments [40–42]. Each rat was placed into a transparent box (426 × 266 × 185 mm) during a habituation period of 60 s. Thereafter, two adhesive labels (13 mm diameter, Tough-Spots®) were applied with equal pressure on each animal’s wrist as bilateral tactile stimuli. The time to contact each paw (“contact time”) and to remove (“removal time”) the adhesive labels were measured with a maximum of 120 s. The contact time is defined as the time it takes for the rat to react to the presence of the adhesive labels. The rat may either shake its paw and/or bring it to its mouth. There were 5 days of pre-testing in order to obtain an optimal level of performance, to limit inter-individual variations and asymmetries. A trial ended when the rat removed both labels or after 3 min.

##### Tapered beam walking test

Motor performance and coordination can be measured with the tapered beam walking test [43]. Prior to surgery, all animals were trained to traverse a 165-cm long elevated, tapering beam with a 2-cm ledge in order to go in their home cage (Campden Instruments, Lafayette, IN, USA) (two trials per day for 5 days). Criterion performance was assessed as the ability to traverse the beam five times in a row without stopping. The day of the test (on days 2, 5, and 7 post-CCI), animals are videotaped while traversing the beam (four trials), and performance was rated on the percentage of contralateral hind limb faults (calculated by dividing the number of foot faults by the total number of steps and multiplying by 100) [44, 45].

##### Limb-use asymmetry test

This procedure was adapted from Schallert et al. [46]. Injured animals use less often their contralateral limbs (injured site) to balance themselves while rearing along the wall of the cylinder [47]. Each animal was placed in a transparent glass cylinder (20 cm diameter and 38 cm height), and its spontaneous activity to explore the vertical surface with its forelimbs was analyzed by videotaping during 3 min. A mirror was placed behind the cylinder at an angle to allow the observer to record forelimb movements when the animal was turned away from the camera. Rats used either both forelimbs or a single one for an exploration. The number of both, right only, or left only explorations was counted. The laterality score was computed as follows: (number of left only − number of right only)/(number of left only + number of right only + number of both). Rats with unilateral lesion prefer to use the limb ipsilateral to the lesion.

#### Effects of EFX treatment on neuroinflammation

##### GFAP and CD68 immunostaining

Forty-height hours after surgery (24 h after the second and last EFX administration), animals were deeply anesthetized with sodium pentobarbital (80 mg/kg) and perfused intracardially with phosphate buffer saline (PBS) at room temperature followed by ice-cold 4 % paraformaldehyde (PFA). Brains were removed and post-fixed in the same fixative for 24 h and soaked in 30 % sucrose solutions. Brains were then frozen and sectioned (50 μm) using a cryostat (Leica CM 1850, Leica, Wetzlar, Germany). Free-floating sections were immunostained for glial fibrillary acidic protein (GFAP), a marker of astrocytic activation, and cluster of differentiation 68 (CD68), a marker of macrophages and activated microglia, as follow: slides were rinsed in tris-buffered saline (TBS, pH 7.4), then incubated for 30 min with hydrogen peroxide to quench endogenous peroxidases, rinsed in TBS-Triton (TBS-T), and pre-incubated 60 min with 5 % blocking serum in TBS-T. Tissue sections were then incubated overnight at 4 °C with a rabbit anti-GFAP antibody (Abcam, Cambridge, UK) diluted (1:5000) or a mouse anti-CD68 antibody (Bio-Rad Laboratories, Hercules, CA, USA) diluted (1:200) in TBS-T containing 2 % normal goat serum (NGS). Slides were rinsed in TBS-T and incubated 30 min with a goat anti-rabbit biotinylated antibody (Abcam) diluted (1:500) or a goat anti-mouse biotinylated antibody (Abcam) diluted (1:500) in TBS-T and 2 % NGS. Slides were then rinsed and processed by using the Vectastain ABC Elite kit (Vector Laboratories, Burlingame, CA, USA) and stained with diaminobenzidine and hydrogen peroxide.

##### Fluoro-jade B staining

Fluoro-jade B (FJB) is a fluorescent marker that sensitively and specifically binds to degenerating neurons [48]. Brain sections were first incubated in absolute ethanol during 3 min, then 70 % ethanol during a minute, and rinsed in distilled water. Sections were then incubated in a solution of 0.06 % potassium permanganate for 15 min, rinsed in distilled water for a minute, and incubated in 0.0004 % solution of FJB (Interchim, Montluçon, France) for 30 min.

##### Image analysis

Slices were mounted on coverslips with Neo-Mount® mounting medium (VWR, Fontenay-sous-Bois, France) before analysis with an optical microscope (Leica DM 4000, Leica) coupled to a camera (Sony XCD-U100CR, Sony, Tokyo, Japan) and to an acquisition system (Archimed, Microvision Instruments, Evry, France). All images were acquired using the same light level by using a 20× (for CD68 and GFAP immunostaining) or 10× (for FJB staining) objective lens. The number of CD68+ cells was counted manually: three sections from each rat were selected for analysis. One section was at the epicenter of the impact (bregma 0 mm) while the other two were either 300 μm rostral or caudal to the epicenter. For GFAP expression, 12 non-consecutive sections were immunostained, representing the area of the lesion (approximately bregma +1.8 mm to bregma −1.8 mm) and quantified. Sections were digitized as grayscale images after background subtraction for quantification of intensity (Image J, NIH, version 1.46). For FJB staining, all sections were observed and photographed with blue excitation light (480 nm). The number of FJB+ cells was counted manually in the contusion margin along the cortex. For all analysis, brain sections from stereotaxically corresponding regions were used in sham-treated rats.

##### Cytokine immunoassay

Cytokine multiplex assay was performed on the cerebral cortex. Brain tissues were harvested and frozen 6 or 12 h post-injury separately from each hemisphere. Tissues were then homogenized in a lysis buffer (RIPA buffer, Abcam) containing protease inhibitor cocktail (Pierce™ protease inhibitor, Thermo Fisher Scientific, Waltham, MA, USA). Tissue homogenates were centrifuged at 13,000 rpm for 20 min at 4 °C. Supernatants were transferred to new tubes and used for analysis. All assays were performed according to the manufacturer’s instructions, in duplicates, and without adjustments to the recommended standard curve dilution. The concentration of 12 cytokines/chemokines (IL-1α, IL-1β, IL-2, IL-4, IL-5, IL-6, IL-10, IL-12, TNF-α, GM-CSF, MCP-1, and MIP-1α) was determinate by Bio-Plex Pro™ rat cytokine assays (catalog number: 171-K1001M, Bio-Rad Laboratories, Hercules, CA, USA) in 500 μg of total protein of brain cortices homogenates (Bradford assay, Bio-Rad Laboratories). Briefly, magnetic beads conjugated with cytokine antibodies were loaded into the wells of a 96-well plate. After washing, standards and samples were added into wells and incubated for 60 min at room temperature on a shaking platform. The beads were washed and incubated with biotinylated detection antibody for 30 min at room temperature. Following the removal of excessive detection antibodies, streptavidin-phycoerythrin conjugate compound was added and allowed to incubate for 10 min at room temperature. Cytokines concentrations were determined by Bio-Plex Manager™ MP software version 6.1 and calculated against the standard curve. Samples with out of range (low) levels were assigned the limit of detection (LOD) (Table 1) of the assay for analysis purposes. The intra-assay percentage coefficient of variation (CV) ranged from 1.28 to 9.48 %.

**Table 1:** Limit of detection (LOD) of the Bio-Plex Pro™ rat cytokine assays (Bio-Rad Laboratories, Hercules, CA, USA)

Targets	Assay sensitivity, pg/mL, LOD (limit of detection)
IL-1α	1
IL-1β	2
IL-2	3
IL-4	1
IL-5	6
IL-6	10
IL-12	0.7
TNF-α	3
GM-CSF	0.6
MIP-1α	12
MCP-1	4

##### Statistical analysis

All data are presented as mean ± standard error of the mean (SEM) and were submitted to a square root transformation to meet the requirements of normality if necessary. Behavioral data were analyzed using three two-way analysis of variance (ANOVA) with repeated measures (RM) with *post hoc *Tukey’s multiple comparison test. The first ANOVA consists in studying the effects of TBI on sensorimotor performances (comparison between sham-vehicle and TBI-vehicle groups) with two factors: time in RM and injury. Then, we did the same type of analysis for the effects of EFX treatment on the TBI group and on the sham-operated one. Significant main or interaction effects were followed by Tukey *post hoc* analyses. Biochemical data were analyzed using a two-way ANOVA (TBI or Sham) x (EFX or Vehicle) with *post hoc* Tukey’s multiple comparison test. Inflammatory load score (ILS) was calculated for each rats as follow: animals with the lowest values for a given cytokine (≤25th percentile) were assigned a 1, between the 26th and the 50th percentile animals were assigned a 2, between the 51th percentile and the 75th percentile animals were assigned a 3, and for the largest values (≥76th percentile), animals were assigned a 4. An ILS for each rat was calculated by summing the quartile rank for each of the pro-inflammatory cytokines. All statistical analyses were performed using SigmaStat® software v 3.5 (Systat Software Inc., San Jose, California, USA). Significance was set at *p* < .05.

## Results

### Etifoxine improves adhesive removal task at 2 days post-CCI

**Fig. 1:**
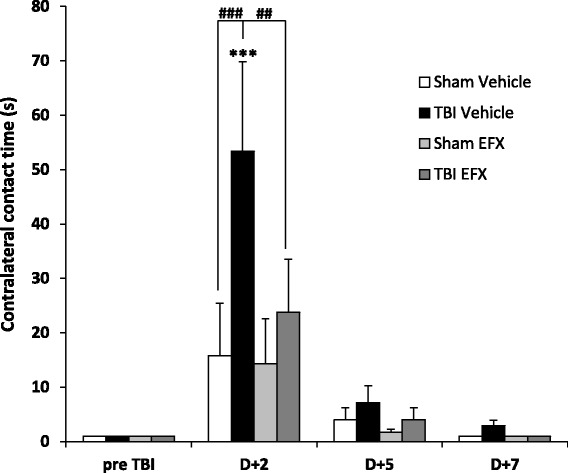
Bilateral adhesive removal test. Mean (+SEM) time to contact the contralateral adhesive in sham-vehicle (*n* = 9), TBI-vehicle (*n* = 10), sham-EFX (*n* = 10), and TBI-EFX (*n* = 8) groups. EFX treatment significantly improved the time to contact on day 2 post-CCI compared with vehicle treatment in TBI animals (^##^ *p* < 0.01). ***p < 0.001 compared to pre-TBI; ^###^ *p* < 0.001 and ^##^ *p* < 0.01 compared to TBI-vehicle

Results showed that before surgery, all groups of rats felt the presence of the adhesive stuck on their wrists very quickly (less than 1 s for each animal). Statistical analyses (two-way RM ANOVA) revealed no significant effect of the injury factor (*F*_1, 18_ = 3.718, *p* = 0.071), a significant effect of time (*F*_3, 54_ = 10.873, *p* < 0.001) and a significant interaction (*F*_3, 54_ = 3.441, *p* = 0.023). *Post hoc* analyses showed that 2 days post-surgery, TBI-vehicle-treated rats needed significantly more time to feel the presence of the adhesive compared to pre-surgery (53.3 ± 16.5 s) (*p* < 0,001). Sham-operated animals did not present any deficit. In the injured group, two-way RM ANOVA showed no effect of treatment (*F*_1, 16_ = 2.208, *p* = 0.157), a significant effect of time (*F*_3, 48_ = 12.826, *p* < 0.001) and no significant interaction (*F*_3, 48_ = 1.954, *p* = 0.133). *Post hoc* analyses showed that EFX treatment significantly reduced the contact time in injured animals 2 days post-surgery (23.7 ± 9.7 s vs. 53.3 ± 16.5 s, *p* = 0.006). No significant differences were observed on day + 5 and day + 7 post-surgery in any groups (Fig. 1).

**Fig. 2:**
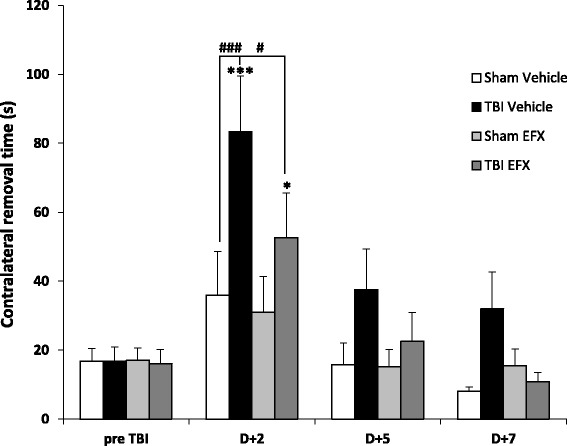
Bilateral adhesive removal test. Mean (+SEM) time to remove the contralateral adhesive in sham-vehicle (*n* = 9), TBI-vehicle (*n* = 10), sham-EFX (*n* = 10), and TBI-EFX (*n* = 8) groups. EFX treatment significantly improved the time to remove the adhesive on day 2 post-CCI compared with vehicle treatment in TBI animals (^#^ *p* < 0.05). ***p < 0.001 and *p < 0.05 compared to pre-TBI; ^###^ *p* < 0.001 and ^#^ *p* < 0.05 compared to TBI-vehicle

Mean removal time was 16.72 ± 1.85 s in all groups before surgery. Two-way RM ANOVA revealed a main effect of injury (*F*_1, 18_ = 6.150, *p* = 0.024), a main effect of time (*F*_3, 54_ = 10.747, *p* < 0.001) and no significant interaction (*F*_3, 54_ = 2.602, *p* = 0.062). *Post hoc* Tukey’s analyses revealed that injured-vehicle-treated animals spent significantly more time to remove the adhesive than sham-vehicle-treated animals at day 2 post-surgery (83.4 ± 16.2 s vs. 35.9 ± 12.6 s, *p* < 0.001). Two-way RM ANOVA showed no significant treatment effect in the injured group (*F*_1, 16_ = 2.744, *p* = 0.117) but a significant effect of time (*F*_3, 48_ = 14.258, *p* < 0.001) and no significant interaction (*F*_3, 48_ = 1.035, *p* = 0.386). *Post hoc* analyses showed that treatment with EFX significantly reduced the time to remove the adhesive in injured animals (52.6 ± 12.9 s vs. 83.4 ± 13.2 s, *p* = 0.043). No significant differences were observed on day + 5 and day + 7 post-surgery in any groups (Fig. 2).

### Etifoxine improves beam walk task at 2 days post-CCI

**Fig. 3:**
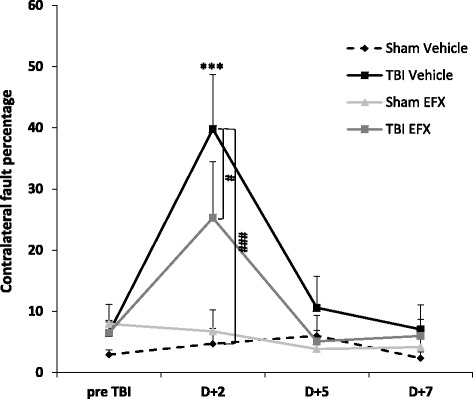
Tapered beam walking test. Mean (+SEM) percentage of faults with the contralateral hind limb on the beam in sham-vehicle (*n* = 9), TBI-vehicle (*n* = 10), sham-EFX (*n* = 10), and TBI-EFX (*n* = 8) groups. Treatment with EFX significantly improved motor performance and coordination on day 2 post-CCI compared to vehicle treatment in TBI animals (^#^ *p* < 0.05). ***p < 0.001 compared to pre-TBI; ^###^ *p* < 0.001 and ^#^ *p* < 0.05 compared to TBI-vehicle

Two-way repeated measures ANOVA revealed that exposure to CCI injury resulted in a main significant effect (*F*_1, 18_ = 10.014, *p* = 0.005), a significant main effect of time (*F*_3, 54_ = 10.624, *p* < 0.001) and a significant interaction (*F*_3, 54_ = 9.084, *p* < 0.001).* Post hoc* analyses showed that 2 days post-injury, TBI-vehicle-treated animals made more foot faults in comparison with sham-vehicle animals (39.8 ± 8.9 vs. 4.7 ± 2.5 %, *p* < 0.001). At day + 5 and day + 7 post-surgery, this impairment is not observed anymore. In the injured groups, two-way RM ANOVA showed no effect of treatment (*F*_1, 16_ = 1.296, *p* = 0.272), a significant main effect of time (F_3, 48_ = 14.666, *p* < 0.001) and no significant interaction (F_3, 48_ = 1.217, *p* = 0.314). *Post hoc* analyses showed that two days of EFX treatment significantly reduced the percentage of contralateral hind limb faults in TBI-EFX animals in comparison with TBI-vehicle-treated animals (25.3 ± 9.2 vs. 39.8 ± 8.9 %, *p* = 0.039) (Fig. 3).

### Etifoxine improves limb-use asymmetry test at 2 days post-CCI

**Fig. 4:**
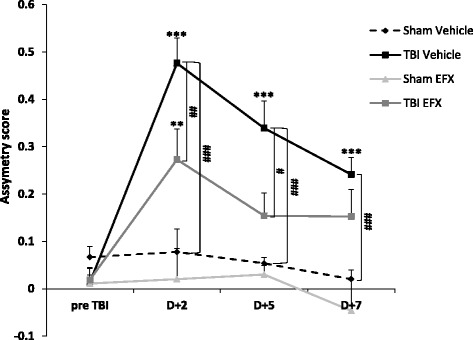
Limb-use asymmetry test. Mean (+SEM) asymmetry score in sham-vehicle (*n* = 9), TBI-vehicle (*n* = 10), sham-EFX (*n* = 10), and TBI-EFX (*n* = 8) groups. Higher scores indicate a greater use of the unimpaired forelimb. Treatment with EFX significantly improved asymmetry score on day 2 (^##^ *p* < 0.01) and day 5 (^#^ *p* < 0.05) post-CCI compared to vehicle treatment in TBI animals. ***p < 0.001 and **p < 0.01 compared to pre-TBI; ^###^ *p* < 0.001, ^##^ *p* < 0.01, and ^#^ *p* < 0.05 compared to TBI-vehicle

Two-way ANOVA with repeated measures showed a significant effect of injury on rats (*F*_1, 18_ = 37.255, *p* < 0.001), a significant effect of time (*F*_3, 54_ = 17.433, *p* < 0.001) and a significant interaction (*F*_3, 54_ = 15.953, *p* < 0.001). *Post hoc* analyses indicated that injured-vehicle-treated rats had higher asymmetry scores at day + 2, day + 5, and day + 7 post-injury compared with sham-vehicle-treated animals (*p* < 0.001). Two-way RM ANOVA showed a significant main effect of EFX treatment in injured rats (*F*_1, 16_ = 7.203, *p* = 0.016), a significant effect of time (*F*_3, 48_ = 25.754, *p* < 0.001) and a significant interaction (*F*_3, 48_ = 3.280, *p* = 0.030). Tukey’s test showed a significant improvement in limb-use asymmetries in EFX-treated animals compared to vehicle-treated animals 2 days post-injury (*p* = 0.003) and 5 days post-injury (*p* = 0.017) (Fig. 4).

### Etifoxine decreases astrogliosis in the injured cortex at 2 days post-CCI

**Fig. 5:**
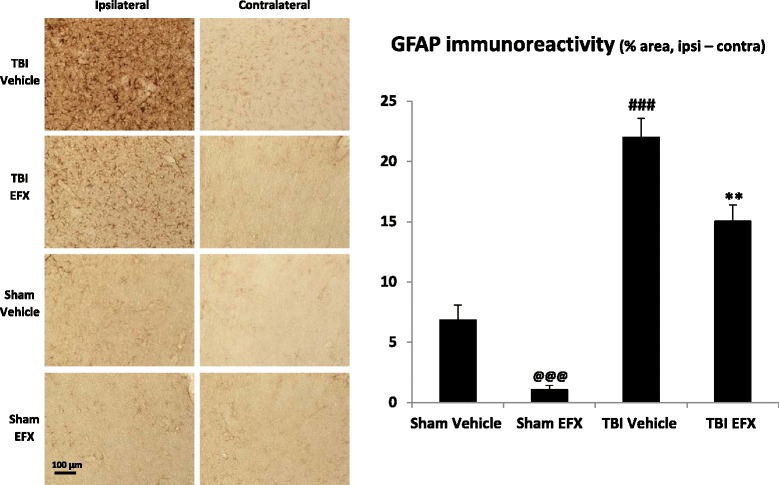
Effect of EFX treatment on CCI-induced astrogliosis in rats 2 days post-injury. EFX (50 mg/kg, i.p.) or its vehicle were administered 30 min after surgery and 24 h after (*n* = 5/group). Astrogliosis was evaluated with GFAP immunostaining. TBI induced an increase in GFAP immunoreactivity in the ipsilateral side of the injury (*p* < 0.001). EFX treatment significantly reduced astrogliosis (*p* < 0.01) in injured animals in comparison with vehicle treatment. Images show representative sections from each group (ipsilateral and contralateral side). Data are mean + SEM. ^@@@^ *p* < 0.001: comparison between sham-vehicle and sham-EFX groups; ^###^ *p* < 0.001: comparison between sham-vehicle and TBI-vehicle groups; **p < 0.01: comparison between TBI-vehicle and TBI-EFX groups

Activated astrocytes were visualized by immunohistochemistry for GFAP. Two-way ANOVA showed a significant main effect of CCI (*F*_1, 223_ = 119.61, *p* < 0.001), a significant main effect of EFX treatment (*F*_1, 223_ = 21.34, *p* < 0.001), and no interaction (*F*_1, 223_ = 0.035, *p* = 0.851). *Post hoc* Tukey’s analyses showed that immunostaining for GFAP revealed evidence of a reactive astrogliosis in regions of the cortex underlying the impact site at day + 2 post-surgery (21.3 ± 1.6 % in the TBI-vehicle group vs. 6.8 ± 1.2 % in the sham-vehicle group, *p* < 0.001). EFX treatment significantly reduced GFAP immunoreactivity in injured animals (15.0 ± 1.3 % in the TBI-EFX group vs. 21.3 ± 1.6 % in the TBI-vehicle group, *p* = 0.001) as well as in sham animals (1.1 ± 0.3 % in the sham-EFX group vs. 6.8 ± 1.2 % in the sham-vehicle group, *p* = 0.001) (Fig. 5). Figure 5 shows the typical star-like hypertrophied aspect of astrocytes in response to injury which is a characteristic marker of the inflammatory cascade induced by CNS trauma and which contributes to later pathology.

### Etifoxine reduces activated microglia/macrophage density in the injured cortex at 2 days post-CCI

**Fig. 6:**
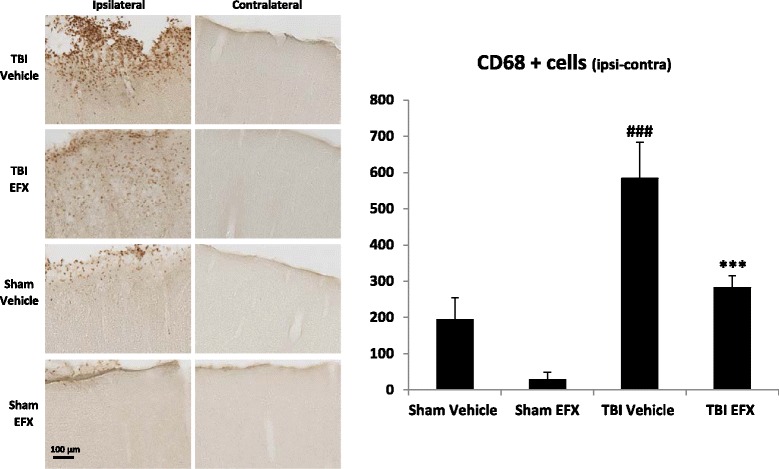
Effect of EFX treatment on CCI-induced microglia/macrophage activation in rats 2 days post-injury. EFX (50 mg/kg, i.p.) or its vehicle were administered 30 min after surgery and 24 h after (*n* = 5/group). Microglia/macrophage activation was evaluated with CD68 immunostaining. TBI induced an increase in CD68 immunoreactivity in the ipsilateral side of the injury (*p* < 0.001). EFX treatment significantly reduced microglia/macrophage activation (*p* < 0.001) in injured animals in comparison with vehicle treatment. Images show representative sections from each group (ipsilateral and contralateral side). Data are mean + SEM. ^###^ *p* < 0.001: comparison between sham-vehicle and TBI-vehicle groups; ***p < 0.001: comparison between TBI-vehicle and TBI-EFX groups

Blood-borne and resident macrophages/activated microglia can be visualized by anti-CD68 staining (Fig. 6). Two-way ANOVA revealed a significant main effect of surgery (*F*_1, 65_ = 24.87, *p* < 0.001), a significant main effect of EFX treatment (*F*_1, 65_ = 13.09, *p* < 0.001), and no interaction (*F*_1, 65_ = 1.099; *p* = 0.298). As shown in Fig. 6, *post hoc* analyses showed a significant increase in CD68+ cells in the cortical contusion margin of rats 2 days post-CCI (584 ± 100 for TBI-vehicle group vs. 194 ± 61 for sham-vehicle group, *p* < 0.001). A significant reduction in the density of activated microglia/macrophages was observed in EFX-treated rats compared with vehicle controls (282 ± 33 in the TBI-EFX group vs. 584 ± 100 in the TBI-vehicle group, *p* < 0.001). No positive staining was observed in the hemisphere contralateral to the impact site.

### Etifoxine reduces neuronal degeneration in the injured cortex at 2 days post-CCI

**Fig. 7:**
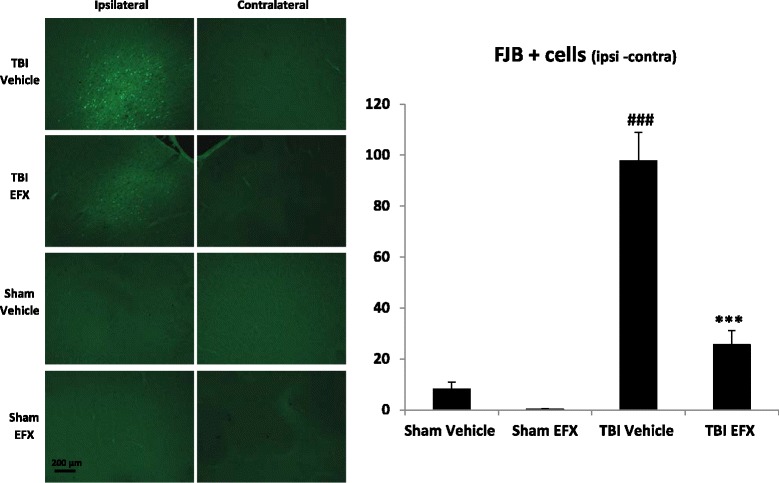
Effect of EFX treatment on CCI-induced neuronal degeneration in rats 2 days post-injury. EFX (50 mg/kg, i.p.) or its vehicle were administered 30 min after surgery and 24 h after (*n* = 5/group). Neuronal degeneration was evaluated with fluoro-jade B (FJB) staining. TBI induced an increase in FJB staining in the ipsilateral side of the injury (*p* < 0.001). EFX treatment significantly reduced neuronal degeneration (*p* < 0.001) in injured animals in comparison with vehicle treatment. Images show representative sections from each group (ipsilateral and contralateral side). Data are mean + SEM. ^###^ *p* < 0.001: comparison between sham-vehicle and TBI-vehicle groups; ***p < 0.001: comparison between TBI-vehicle and TBI-EFX groups

Statistical analyses revealed a significant main effect of surgery (*F*_1, 297_ = 51.439, *p* < 0.001), a significant main effect of EFX treatment (*F*_1, 297_ = 20.042, *p* < 0.001), and a significant interaction (*F*_1, 297_ = 10.521, *p* = 0.001). *Post hoc* analyses showed that FJB+ cells with neuronal morphology were evident 2 days after CCI in the cortical contusion margin but not in the contralateral hemisphere. Ipsilateral minus contralateral FJB+ cells counting showed that neuronal degeneration was significantly more important in TBI-vehicle group compared with sham-vehicle group (98 ± 11 for TBI-vehicle group vs. 8 ± 3 for sham-vehicle group, *p* < 0.001). Etifoxine treatment significantly reduced the number of FJB+ cells in CCI rats compared with vehicle treatment (26 ± 5 for TBI-EFX group vs. 98 ± 11 for TBI-vehicle group, *p* < 0.001) (Fig. 7).

### Etifoxine reduces cortical level of pro-inflammatory cytokines

**Fig. 8:**
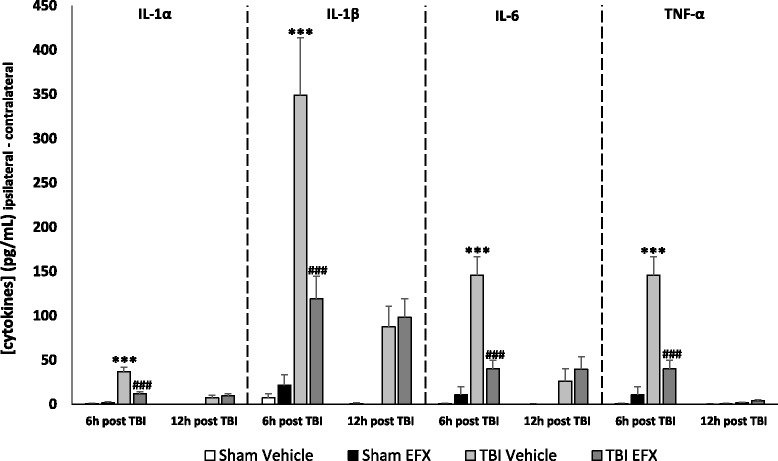
Cortical cytokine levels at 6 and 12 h post-CCI. The pro-inflammatory cytokines IL-1α, IL-1β, Il-6, and TNF-α were significantly elevated 6 h after CCI in the injury and margin regions compared to sham animals (***p < 0.001 for all groups). Statistical analyses showed no difference at 12-h time-point. Six hours after CCI, EFX treatment (50 mg/kg, i.p., 30 min after injury) significantly reduced IL-1α, IL-1β, IL-6, and TNF-α peaks (^###^ *p* < 0.001 for all groups). ***p < 0.001: comparison between sham-vehicle and TBI-vehicle groups; ^###^ *p* < 0.001: comparison between TBI-vehicle and TBI-EFX groups

Different studies showed that the temporal change of several inflammatory cytokines peaked at 6 h after CCI [3, 49]. We therefore selected this time-point for evaluating EFX treatment on cytokine/chemokine expression. There are no differences within contralateral sites of injury between groups. Two-way ANOVA revealed statistical differences for four pro-inflammatory cytokines, IL-1α, IL-1β, IL-6, and TNF-α, and two chemokines: MCP-1 and MIP-1α. *Post hoc* testing showed that there was a significant increase in IL-1α, IL-1β, IL-6, TNF-α, MCP-1, and MIP-1α levels in the ipsilateral cortices to the injury site of TBI-vehicle-treated group in comparison with sham-vehicle-treated animals (*p* < 0.001). Statistical analyses also showed that EFX treatment significantly reduced this increase in pro-inflammatory cytokines and chemokines levels in the injured group compared to vehicle treatment (*p* < 0.001 for IL-1α, IL-1β, IL-6, and TNF-α, *p* < 0.05 for MCP-1 and MIP-1α). Data are summarized in Table 2. Because multiple testing across the analytes may not be fully accounted for by multiple test correction within each analyte, resulting in type I errors, we have also performed an inflammatory load score (ILS) analysis (Table 3). Two-way ANOVA revealed an overall effect of TBI on inflammation (*F*_1,15_ = 15.46, *p* = 0.001), an overall effect of EFX treatment (*F*_1, 15_ = 11.50, *p* = 0.004) on ILS, and no interaction (*F*_1, 15_ = 3.584, *p* = 0.078). *Post hoc* analysis showed that injured animals have a higher ILS compared to sham animals (*p* < 0.001) and that EFX treatment significantly reduced ILS in injured animals (*p* = 0.003) (see Table 3). We also tested the levels of pro-inflammatory cytokines IL-1α, IL-1β, IL-6, and TNF-α 12 h after surgery in order to study their evolution of expression. At 6 h time-point, the profound increase in pro-inflammatory cytokines associated with trauma was significantly reduced by EFX treatment (*p* < 0.001) compared with vehicle-treated rats to such an extent that these values were not different from those for the 12 h time-point (Fig. 8). We did not find any modifications of other tested cytokines in injured animals compared to sham animals (data not shown).

**Table 2:** Concentrations of cytokines/chemokines in the injured cortex at 6 h post-CCI or sham-operation in rats treated with EFX or Vehicle control

	Sham-vehicle (pg/mL)		Sham-EFX (pg/mL)		TBI-vehicle (pg/mL)		TBI-EFX (pg/mL)		TBI-vehicle vs. TBI-EFX
	Ipsilateral	Contralateral	Ipsilateral	Contralateral	Ipsilateral	Contralateral	Ipsilateral	Contralateral	% decrease (ipsilateral side)
Pro-inflammatory cytokines									
IL-1α	5.75 ± 0.87	5.17 ± 0.22	5.53 ± 0.81	3.83 ± 0.88	40.41 ± 5.14***	3.27 ± 0.63	13.55 ± 2.65**	1.32 ± 0.77	33
IL-1β	45.14 ± 2.89	37.68 ± 2.43	56.58 ± 9.43	34.77 ± 3.25	382.88 ± 64.66***	33.98 ± 1.57	148.97 ± 25.50**	29.82 ± 1.72	39
IL-2	26.47 ± 0.75	24.84 ± 0.70	23.48 ± 0.50	22.89 ± 1.57	27.55 ± 4.42	21.55 ± 0.62	23.44 ± 1.64	20.46 ± 0.64	ns
IL-6	2.65 ± 0.33	1.76 ± 0.23	14.00 ± 8.89	3.21 ± 0.71	150.18 ± 21.25***	4.34 ± 0.64	41.97 ± 10.25**	1.86 ± 0.51	28
IL-12	8.87 ± 0.29	8.54 ± 0.38	7.50 ± 0.24	7.50 ± 0.95	7.41 ± 0.26	6.95 ± 0.38	6.95 ± 0.20	7.10 ± 0.20	ns
TNF-α	5.34 ± 0.35	1.30 ± 0.14	5.93 ± 0.36	4.48 ± 0.84	16.34 ± 1.71***	5.24 ± 0.62	9.91 ± 1.28**	5.91 ± 0.70	60
GM-CSF	2.49 ± 0.67	2.24 ± 0.49	2.06 ± 0.26	1.52 ± 0.29	1.20 ± 0.22	1.15 ± 0.17	1.07 ± 0.27	1.36 ± 0.14	ns
Anti-inflammatory cytokines									
IL-4	2.86 ± 0.13	2.50 ± 0.08	3.20 ± 0.53	2.85 ± 0.51	4.19 ± 0.78	2.54 ± 0.05	3.65 ± 0.72	2.32 ± 0.13	ns
IL-5	11.22 ± 0.24	10.74 ± 0.24	7.93 ± 0.31	6.72 ± 1.71	9.34 ± 0.63	9.24 ± 0.89	9.30 ± 1.18	9.00 ± 1.13	ns
IL-10	25.99 ± 1.60	24.13 ± 1.67	19.94 ± 2.04	19.66 ± 4.35	35.29 ± 4.98	17.52 ± 2.82	28.48 ± 2.45	23.12 ± 0.83	ns
Macrophage-attracting chemokines									
MIP-1α	1.71 ± 1.01	0.04 ± 0.04	2.64 ± 1.3	0.00 ± 0.00	90.42 ± 25.47***	0.00 ± 0.00	36.99 ± 4.51*	0.24 ± 0.21	41
MCP-1	7.50 ± 1.97	4.30 ± 0.63	12.82 ± 5.16	4.25 ± 0.64	93.82 ± 21.66***	5.53 ± 0.97	53.00 ± 7.45*	6.34 ± 1.55	56

Six hours post TBI, ipsilateral and contralateral cortices were analyzed with the Bio-Plex Pro™ rat cytokine assays for IL-1α, IL-1β, IL-2, IL-4, IL-5, IL-6, IL-10, IL-12, TNF-α, GM-CSF, MIP-1α, and MCP-1

Data (*n* = 5/groups) represents mean ± SEM. *p* < 0.05 or less were considered statistically significant. Two-way analysis of variance was performed, followed by Tukey *post hoc* test

*NS* not significant

***p < 0.001: comparison between sham-vehicle and TBI-vehicle groups in the ipsilateral side; **p < 0.001 and *p < 0.05: comparison between TBI-vehicle and TBI-EFX groups in the ipsilateral side

**Table 3:** Inflammatory load score (ILS) of each group of rats (sham-vehicle, sham-EFX, TBI-vehicle, TBI-EFX, *n* = 5/groups) measured in the ipsilateral side of the lesion 6 hours post TBI

	Sham			TBI		
	Vehicle	EFX	*p* value	Vehicle	EFX	*p* value
Mean ILS (SEM)	2.371 (0.21)	2.114 (0.15)	0.292	3.371 (0.15)	2.464 (0.16)	0.003

Ipsilateral cortices were analyzed with the Bio-Plex Pro™ rat cytokine assays. The cytokines used for the score were IL-1α, IL-1β, IL-2, IL-6, IL-12, TNF-α, and GM-CSF. Data represents mean (SEM)

## Discussion

Our results showed that TBI model induced by CCI caused sensorimotor deficits in rats measured in three behavioral tasks: bilateral adhesive removal test, tapered beam walk test, and limb-use asymmetry test. An administration of EFX 30 min after the injury, followed by a second injection after 24 h, significantly improved functional impairments. This protective effect was associated with reduced neuroinflammation, astrogliosis, and neuronal degeneration.

Following TBI, there is a wide range of cellular and biochemical pathological events resulting in neuron and glial cells death. Among these events, neuroinflammation is characterized by the activation of resident astrocytes and microglia and by the infiltration of leukocytes into the injury site. These events lead to the release of inflammatory cytokines and neurotoxic molecules which contribute to progressive cell death and functional impairments [50].

Our results suggest that EFX may reduce delayed neuronal death that contributes to neurological deficits [51] and promote survival in the injury border zone. This hypothesis is supported by the observations that EFX-treated animals showed fewer degenerative neurons than vehicle-treated rats as evaluated by FJB staining.

Among the different mechanisms underlying delayed neuronal loss, post-traumatic neuroinflammation is a major factor. Activation of microglia is one of the central inflammatory responses after TBI [52]. Recent studies showed that brain inflammation has been associated with polarization of microglia and macrophages (M1 and M2 responses) [53–56]. M1 response is an activation of microglia/macrophages that produces pro-inflammatory substances such as IL-1β, IL-6, and TNF-α [56]. The M2 response is induced by anti-inflammatory cytokines such as IL-4 and IL-10 [53]. Inflammation is therefore an important tool for controlling TBI effects and enhancing recovery after injury. Thus, the suppression of M1 and/or the up-regulation of M2 response could be used to prevent secondary injury and promote repair processes in the brain. In a study using male Sprague-Dawley rats, Ansari showed an upregulation of pro-inflammatory cytokines (TNF-α, IL-1β, and IL-6) expression 6 h post-CCI and an upregulation of anti-inflammatory cytokines such as IL-4 and IL-10 but only 24 h post-injury [57]. These results support other studies showing that early after injury, M1 predominate over M2 response [54, 58, 59]. These observations are in accordance with our results showing an upregulation of pro-inflammatory cytokines such as TNF-α, IL-1α, IL-1β, and IL-6 early after injury but no upregulation of anti-inflammatory cytokines such as IL-4 and IL-10 6 h post-injury. Pro-inflammatory cytokines secreted by activated microglia also secrete chemokines that recruit additional inflammatory cells to the injury site and free radicals that are cytotoxic to neurons and can contribute to neurodegeneration after TBI [60, 61]. Elevated levels of pro-inflammatory cytokines have been detected in the serum and cerebrospinal fluid of TBI patients [62, 63] and in brain parenchyma of animals with experimental brain injuries [64–67]. The role of these pro-inflammatory cytokines has remained controversial, but several studies showed that suppressing their expression may reduce tissue damage and brain edema and also improve functional consequences [2, 68–78]. We demonstrated that acute administration of EFX significantly reduced TBI-induced up-regulation of pro-inflammatory cytokines. Since TNF-α, IL-1β, and IL-6 are major pro-inflammatory cytokines that mediate blood brain barrier disruption, edema, and programmed cell death resulting in loss of neurons [3, 71, 79–83], we can hypothesized that EFX may exerts its neuroprotective actions, at least in part, by suppressing the expression of these cytokines which are detrimental in the acute post-traumatic period.

Experimental and clinical data show that GFAP and other intermediate filaments such as nestin and vimentin are considerably upregulated following TBI [84–86]. In our study, 2 days after TBI, there was a significant increase in GFAP immunoreactivity in the perimeter of the cortical lesion and in the subcortical white matter, reflecting increased astrocytic activation in the cortex ipsilateral to the injury site. This observation is in accordance with other studies [64, 85]. Two days of EFX treatment significantly reduced this astrocytic activation. This is important to note because astrocytic activation increases the production of pro-inflammatory cytokines, reactive oxygen species, excitatory amino acids, and nitrogen oxides and can also induce blood brain barrier disruptions that induce immune cell infiltration [87–89]. GFAP also promotes scarring and glial proliferation which is deleterious on neuronal regeneration and recuperation [90].

Our results are in accordance with other studies that show that TSPO ligands exert neuroprotective effects and reduce neural inflammation [26, 28–30]. Girard et al. indeed showed that EFX treatment decreased messenger RNA (mRNA) expression of pro-inflammatory cytokines TNF-α, Il-1β, and IL-6 and improved functional recovery in a model of peripheral nerve injury in rats and also had beneficial effects on axonal regrowth and on macrophage response [30, 32]. Ravikumar et al. showed that EFX treatment reduced lipopolysaccharide (LPS)-induced IL-1β and IL-6 release from rat primary astrocytes in a concentration-dependent manner [91]. Daugherty et al. also showed a decrease in pro-inflammatory cytokines after EFX treatment in a model of multiple sclerosis [92].

Etifoxine, which is a TSPO ligand, has been shown to be a potent enhancer of neurosteroidogenesis and to induce the synthesis of pregnenolone, progesterone, and allopregnanolone [18, 20, 93] which have demonstrated neuroprotective properties. Progesterone treatment following TBI in rats has indeed been demonstrated to reduce cerebral edema, cell death mediators expression, inflammatory response, reactive gliosis, and necrosis and to improve functional recovery [94–100]. In their studies, He at al. [8, 101] demonstrated that allopregnanolone significantly reduced neuronal loss and enhanced cognitive and behavioral recovery in a model of TBI in rats. These results suggest that EFX, a TSPO ligand, may protect neuronal function by inducing neurosteroids biosynthesis.

In addition to its action on TSPO receptor, EFX exerts a direct effect on GABA_A_ receptor as a positive allosteric modulator [10]. This is of interest because a significant decrease in inhibitory synaptic transmission has been shown in the basolateral amygdala of rats subjected to CCI, associated with a decrease of surface expression of GABA_A_ receptors [102]. Furthermore, glutamatergic projections from the mediodorsal thalamic nucleus and cholinergic projections from the nucleus basalis magnocellularis to the prefrontal cortex are known to be modulated by GABA transmission [103, 104] and are highly sensitive to TBI [8, 94]. Excessive release of glutamate and acetylcholine following TBI causes excitotoxicity that could thus be prevented by direct action of EFX on inhibitory GABA_A_ receptors and its indirect action on the synthesis of allopregnanolone, which also potentiate GABA transmission [105, 106].

## Conclusions

EFX treatment after TBI considerably improves functional outcomes. This effect is associated with reductions in neuronal degeneration, in glial scar formation and in microglial activation. Neuroinflammation is a major secondary injury mechanism that provides later neurodegeneration and neurologic impairments associated with TBI. We can hypothesize that functional and histological improvement of EFX-treated animals is due to its modulation of early inflammation mechanisms. These results are interesting because pre-clinical studies which combine both neurobehavioral and histological evaluations may improve the predictive value of animal models for clinical efficacy with novel neuroprotective agent [50]. In addition, the beneficial neuroprotective effects of EFX at an earliest stage of TBI might attenuate other deleterious effects such as anxiety disorders and memory disturbances which can appear several weeks later [84, 85].

## Abbreviations

TBI: Traumatic brain injury
EFX: Etifoxine
GABA: Gamma-aminobutyric acid
TSPO: Translocator protein 18 kDa
kDa: Kilodalton
CNS: Central nervous system
CCI: Controlled cortical impact
PBS: Phosphate buffer saline
PFA: Paraformaldehyde
GFAP: Glial fibrillary acidic protein
CD68: Cluster of differentiation 68
PH: Potential of hydrogen
TBS: Tris-buffered saline
TBS-T: Tris-buffered saline-Triton
NGS: Normal goat serum
FJB: Fluoro-jade B
RPM: Revolution per minute
IL-1α: Interleukin-1 alpha
IL-1β: Interleukin-1 beta
IL-2: Interleukin-2
IL-4: Interleukin-4
IL-5: Interleukin-5
IL-6: Interleukin-6
IL-10: Interleukin-10
IL-12: Interleukin-12
TNF-α: Tumor necrosis factor alpha
GM-CSF: Granulocyte macrophage-colony stimulating factor
MCP-1: Monocyte chemotactic protein 1
MIP-1α: Macrophage inflammatory protein 1 alpha
SEM: Standard error of the mean
ANOVA: Analysis of variance
RM: Repeated measure
LOD: Limit of detection
ILS: Inflammatory load score
LPS: Lipopolysaccharide
